# Septic Encephalopathy Characterized by Acute Encephalopathy with Biphasic Seizures and Late Reduced Diffusion and Early Nonconvulsive Status Epilepticus

**DOI:** 10.1155/2016/7528238

**Published:** 2016-03-09

**Authors:** Hiroshi Yamaguchi, Tsukasa Tanaka, Azusa Maruyama, Hiroaki Nagase

**Affiliations:** ^1^Department of Emergency and Critical Care Medicine, Hyogo Prefectural Kobe Children's Hospital, 1-1-1 Takakuradai, Suma-Ku, Kobe, Hyogo 654-0081, Japan; ^2^Department of Neurology, Hyogo Prefectural Kobe Children's Hospital, 1-1-1 Takakuradai, Suma-Ku, Kobe, Hyogo 654-0081, Japan

## Abstract

Infection, whether viral or bacterial, can result in various forms of brain dysfunction (encephalopathy). Septic encephalopathy (SE) is caused by an excessive immune reaction to infection, with clinical features including disturbed consciousness and seizures. Acute encephalopathy with biphasic seizures and late reduced diffusion (AESD) is usually accompanied by viral infection in children and is characterized by biphasic seizures and impaired consciousness. The initial neurologic symptom of AESD is typically a febrile seizure that frequently lasts longer than 30 minutes. However, the possible forms this seizure takes are unclear. For example, it is unknown if nonconvulsive status epilepticus (NCSE) could be an early seizure symptomatic of AESD. In addition, thus far no cases of combined SE and AESD have been reported. Here, we describe the first reported case of SE with AESD that notably demonstrated NCSE as an early seizure.

## 1. Introduction

Both bacterial and viral infections can induce forms of brain dysfunction (encephalopathy) whose symptoms frequently include seizures of one type or another. Septic encephalopathy (SE) is a brain dysfunction characterized by clinical, electrophysiological, or biochemical criteria and is considered to be primarily due to an excessive immune reaction to infection [1]. The main clinical features of SE are disturbances of consciousness, impaired cognitive function, and seizures [1]. The pathophysiology of SE is still poorly understood, although many mechanisms of its development have been proposed. These include oxidative stress, increased cytokine and proinflammatory factor levels, disturbances in cerebral circulation, injury to the brain's vascular endothelium, altered neurotransmitter levels, and bacterial endotoxins leaking through the blood-brain barrier [1]. Estimates suggest that 8–70% of the patients with diagnosed sepsis exhibit symptoms of encephalopathy [2].

Another infection-related encephalopathic disorder is acute encephalopathy with biphasic seizures and late reduced diffusion (AESD). AESD is characterized by biphasic seizures and impaired consciousness, preceded most often by viral infection. These symptoms are followed by reduced diffusion in the subcortical white matter upon magnetic resonance imaging (MRI) that is typically observed between days 3 and 9 after the clinical onset [3]. Typically, the initial neurologic symptom of AESD is a febrile seizure that usually lasts longer than 30 minutes [4, 5]. While it is possible that other types of early seizures can represent AESD, to the best of our knowledge no reports of nonconvulsive status epilepticus (NCSE) as such a seizure exist.

To the best of our knowledge, no cases of SE in which AESD was also present have been reported. However, we report here a case of a 3-year-old Japanese boy who after* Streptococcus pneumoniae* (*S. pneumoniae*) bacteremia developed SE with both the clinical and the radiological features of AESD. Notably, this is apparently the first reported case in which NCSE represented the early seizure symptom of AESD.

## 2. Case Report

A 3-year-old Japanese boy was admitted to our hospital presenting with a high fever and shivering. His past medical history included congenital asplenia syndrome, an esophageal hiatal hernia after cardioplasty, and a single cardiac atrium and ventricle after a Fontan procedure. These conditions were controlled by aspirin, warfarin, diuretics, and home oxygen therapy (0.5 L/min oxygen at night). His premorbid activities of daily living (ADL) were appropriate for his age, including the ability to speak in complete sentences and the ability to walk and eat without assistance. He also had no history of hypoxic encephalopathy.

On admission, he showed disturbance of consciousness (Glasgow Coma Scale (GCS) 10 (E3, V3, and M4)). Vital signs were as follows: temperature: 40.2°C; blood pressure (BP): 80/40 mmHg; heart rate (HR): 144 bpm; respiratory rate: 56/min; and oxygen saturation: 96% (0.5 L/min oxygen). Shortly after admission, the patient suffered a tonic-clonic convulsion for 30 seconds, which subsided without treatment. Laboratory data showed leukocytosis (white blood cell count 21,600/*μ*L) but were otherwise normal. Cerebrospinal fluid (CSF) analysis was also normal, and a CSF culture was negative. We diagnosed him with SE and started cefotaxime (CTX; 300 mg/kg/day) for an infection of undetermined origin.

**Figure 1:**
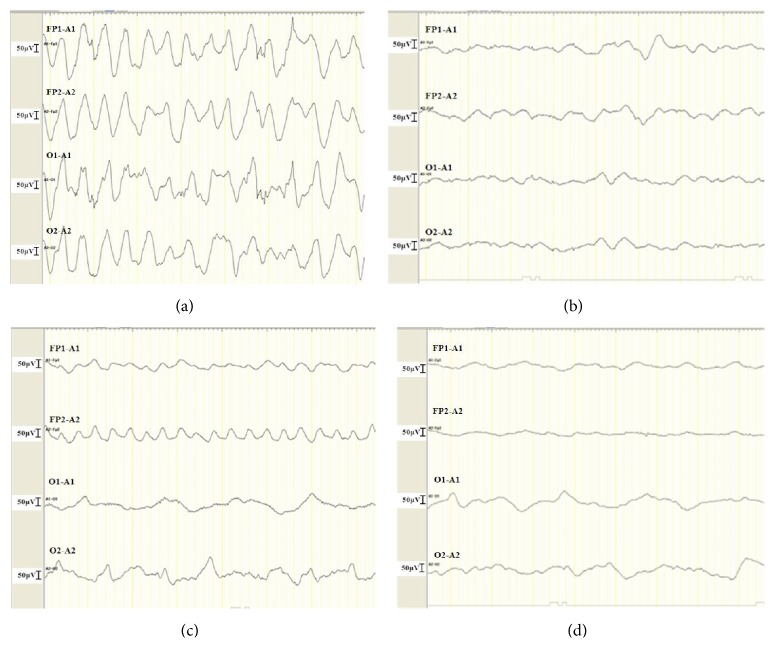
Encephalographic (EEG) findings before and after ictal events on day 1 ((a) and (b)) and day 5 after admission ((c) and (d)), respectively. EEG was digitally recorded using four channels (Fp1-A1, Fp2-A2, O1-A1, and O2-A2) according to the International 10–20 system.

After admission, he continued to be drowsy, and, by 4 hours after admission, his mental status had deteriorated to GCS 6 (E1, V2, and M3) with mumbling. We then started electroencephalography (EEG), which revealed rhythmical, diffuse high-voltage slow activity (Figure 1(a)), which we diagnosed as NCSE. Both electrical seizures and nonconvulsive seizures such as ocular deviation continued intermittently without full recovery of consciousness, despite the administration of midazolam and fosphenytoin. The seizures were finally controlled by phenobarbital (20 mg/kg IV) ten hours after admission (Figure 1(b)). However, the NCSE, high fever (>38°C), and hemodynamic instability (systolic BP: 80–100 mmHg, HR: 150–180 bpm) continued. Treatment with volume load and vasopressor therapy (dopamine drip was up to 6 mcg/kg/min) was initiated, and within several hours the hemodynamics and urine output were restored to within normal range. Although the intermittent seizures without recovery of consciousness were suggestive of refractory status epilepticus, we were reluctant to initiate barbiturate coma therapy because of the hemodynamic instability. His blood culture on admission was positive for* S. pneumoniae*, so we then diagnosed him with sepsis due to* S. pneumoniae*. The next day, his hemodynamic parameters continued to improve with vasopressor therapy (dopamine drip 4.5 mcg/kg/min). At this point, neither electrical nor nonconvulsive seizures developed, so anticonvulsive therapy was discontinued. However, the patient was still drowsy, with a GCS of 6 (E1, V2, and M3).

**Figure 2:**
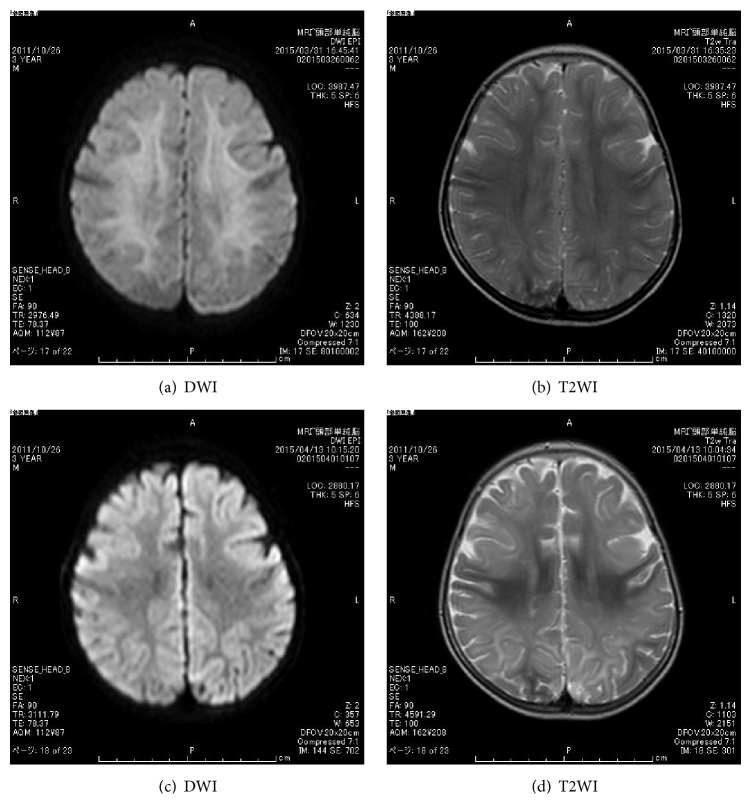
Magnetic resonance imaging (MRI) findings. Both diffusion-weighted imaging (DWI) ((a) and (c)) and T2-weighted imaging (T2WI) ((b) and (d)) were performed. MRI performed on day 8 showed hyperintensity in the deep subcortical white matter ((a) and (b)). The hyperintensity on DWI resolved (c), but diffuse atrophy was noted.

On day 3 after admission, we discontinued vasopressor therapy. Antimicrobial susceptibility testing showed penicillin-sensitive* Streptococcus pneumoniae* (PSSP), so his antibiotics were changed to aminobenzyl penicillin (ABPC; 300 mg/kg/day), which was continued for 14 days. His altered state of consciousness also gradually improved to GCS7 (E1, V2, and M4) on day 3 and GCS9 (E2, V2, and M5) on days 4 and 5, respectively. No seizures were observed from days 3 to 5. On day 6 after admission, the patient had a brief seizure that included rolling of the eyes and apnea; an EEG showed rhythmical, right frontal-dominant slow activity (Figure 1(c)), and his mental status deteriorated again to GCS 6 (E1, V2, and M3). We restarted the treatment with fosphenytoin followed by phenobarbital. Despite these treatments, nonconvulsive and/or electrical seizures were intermittently observed without full recovery of consciousness for 12 hours. On day 7, we started high-dose phenobarbital for refractory NCSE, at daily doses of up to 20 mg/kg IV that were tapered by 50% every other day until day 12. This treatment successfully controlled the seizures (Figure 1(d)). Diffusion-weighted magnetic resonance images (DWI) taken 3 days after the second onset of seizures (day 8 after admission) revealed hyperintensity in the subcortical white matter (bright tree appearance) (Figures 2(a) and 2(b)), which subsequently resolved by day 21 after admission (Figures 2(c) and 2(d)). Thereafter, his clinical condition stabilized, including gradual recovery of consciousness (GCS 11 (E4, V2, and M5)), although he could not walk without support nor speak a meaningful word. He was finally discharged from our hospital 50 days after admission and he returned for a follow-up visit.

## 3. Discussion

During sepsis, the central nervous system (CNS) is one of the first organs damaged, and this clinically leads to SE. Our case of SE followed a clinical course that included biphasic seizures and worsening consciousness as has been described for AESD. Furthermore, MRI studies performed on days 8 and 21 showed the reduced diffusion characteristic of AESD. Given these similarities, we diagnosed him with SE accompanied by AESD. Viral infections such as influenza or HHV-6, or adverse effects after vaccination, have been reported as the main etiologies of AESD [5]. Although bacterial infection is a very rare cause of AESD, a case that was associated with* S. pneumoniae* meningoencephalitis was recently reported [6]. The most unusual aspect of our case was that the patient developed sepsis from* S. pneumoniae *bacteremia; however, we could not find any evidence of meningitis. Therefore, we believe the neuronal injury in this patient was not the result of bacterial circulation, but rather excitotoxicity related to the pathology of AESD [3, 5]. In fact, several mechanisms for brain injury during sepsis have been reported [1, 2], including inflammation that activates excitotoxicity and oxidative stressors which may further aggravate SE and result in neuronal dysfunction [7]. Furthermore, it is possible that the initial low BP in this patient will have led to hypoperfusion and hypoxic ischemia. However, the patient did not have significant hypotension, and his hemodynamic status was successfully controlled, so we do not believe this to be the case.

Our case also revealed NCSE as an early seizure that might lead to AESD. Though the early seizure in AESD usually lasts longer than 30 minutes [4, 5], Takanashi et al. identified some patients with AESD with brief early febrile seizures followed by secondary seizures and disturbance of consciousness on days 4 to 6 after admission [8]. It is unknown whether these patients also had nonconvulsive seizures. Here, the patient had a brief convulsive seizure, after which NCSE was identified through continuous EEG monitoring. NCSE is the diagnosis for encephalopathy caused by continuous epileptic activity on an EEG. It is a well-known cause of morbidity and mortality in critically ill neonates and adults [9, 10]. Recent prospective studies that focused on critically ill children found that NCSE is also common in critically ill children with acute encephalopathy [11, 12]. The impact of NCSE on neurological outcomes is unclear, although evidence suggests that NCSE could be an independent risk factor for hippocampal atrophy [13].

As for the second seizure, it is possible, but unlikely, that the biphasic behavior of this patient could be related to the sudden withdrawal of anticonvulsants. Sudden withdrawal of antiepileptic drugs usually appears within a few days after discontinuation of the drugs and is caused by continuous dosing of an anticonvulsant for a long period. We used anticonvulsants for the early seizures, on only the first day of admission, whereas the biphasic seizure reappeared 5 days later. In addition, the MRI results noted on day 8 are uncommonly found in situations of withdrawal of antiepileptic drugs. Therefore, the biphasic behavior of this patient is not likely to be the result of the sudden withdrawal of antiepileptic drugs.

Our case demonstrates the importance of continuous EEG monitoring for patients with disturbed consciousness even after the convulsive seizures have disappeared, particularly in cases of acute encephalopathy. In these cases, there is the possibility that the early NCSE will lead to mental deterioration and brain damage and culminate in a second seizure. In addition, our case highlights targeted temperature management and/or barbiturate coma therapy for preventing biphasic seizures and neurologic sequelae. These treatments are effective in preventing the damage caused by refractory febrile convulsive status epilepticus or acute encephalopathy [14, 15] and could likely prevent secondary seizures as well. Unfortunately, because of the hemodynamic instability, we could not perform barbiturate coma therapy as we normally would have in such a case as this.

In conclusion, we describe the first reported case of SE with clinical characteristics of AESD with NCSE as an early seizure. Further studies will be needed to determine the exact relationship between SE and AESD.

## References

[1] Ziaja M. (2013). Septic encephalopathy. *Current Neurology and Neuroscience Reports*.

[2] Kafa I. M., Bakirci S., Uysal M., Kurt M. A. (2010). Alterations in the brain electrical activity in a rat model of sepsis-associated encephalopathy. *Brain Research*.

[3] Takanashi J.-I. (2009). Two newly proposed infectious encephalitis/encephalopathy syndromes. *Brain and Development*.

[4] Mizuguchi M., Yamanouchi H., Ichiyama T., Shiomi M. (2007). Acute encephalopathy associated with influenza and other viral infections. *Acta Neurologica Scandinavica*.

[5] Takanashi J., Oba H., Barkovich A. J. (2006). Diffusion MRI abnormalities after prolonged febrile seizures with encephalopathy. *Neurology*.

[6] Kuwata S., Senzaki H., Urushibara Y. (2012). A case of acute encephalopathy with biphasic seizures and late reduced diffusion associated with *Streptococcus pneumoniae* meningoencephalitis. *Brain and Development*.

[7] Dal-Pizzol F., Tomasi C. D., Ritter C. (2014). Septic encephalopathy: does inflammation drive the brain crazy?. *Revista Brasileira de Psiquiatria*.

[8] Takanashi J., Tsuji M., Amemiya K., Tada H., Barkovich A. J. (2007). Mild influenza encephalopathy with biphasic seizures and late reduced diffusion. *Journal of the Neurological Sciences*.

[9] Shneker B. F., Fountain N. B. (2003). Assessment of acute morbidity and mortality in nonconvulsive status epilepticus. *Neurology*.

[10] Pisani F., Sisti L., Seri S. (2009). A scoring system for early prognostic assessment after neonatal seizures. *Pediatrics*.

[11] Greiner H. M., Holland K., Leach J. L., Horn P. S., Hershey A. D., Rose D. F. (2012). Nonconvulsive status epilepticus: the encephalopathic pediatric patient. *Pediatrics*.

[12] Abend N. S., Gutierrez-Colina A. M., Topjian A. A. (2011). Nonconvulsive seizures are common in critically ill children. *Neurology*.

[13] Vespa P. M., McArthur D. L., Xu Y. (2010). Nonconvulsive seizures after traumatic brain injury are associated with hippocampal atrophy. *Neurology*.

[14] Nishiyama M., Tanaka T., Fujita K., Maruyama A., Nagase H. (2015). Targeted temperature management of acute encephalopathy without AST elevation. *Brain and Development*.

[15] Nagase H., Nishiyama M., Nakagawa T., Fujita K., Saji Y., Maruyama A. (2014). Midazolam fails to prevent neurological damage in children with convulsive refractory febrile status epilepticus. *Pediatric Neurology*.

